# Schnurri-3 inhibition rescues skeletal fragility and vascular skeletal stem cell niche pathology in a mouse model of osteogenesis imperfecta

**DOI:** 10.21203/rs.3.rs-3153957/v1

**Published:** 2023-07-26

**Authors:** Ren Xu, Na Li, Baohong Shi, Zan Li, Jie Han, Jun Sun, Alisha Yallowitz, Seoyeon Bok, Shuang Xiao, Zouxing Wu, Yu Chen, Yan Xu, Tian Qin, Zhiming Lin, Haiping Zheng, Rong Shen, Matthew Greenblatt

**Affiliations:** State Key Laboratory of Cellular Stress Biology, School of Medicine, Faculty of Medicine and Life Sciences, Xiamen University; Xiamen University; Xiamen University; First Affiliated Hospital of Zhejiang University; Xiamen University; Weill Cornell Medicine, Cornell University; Cornell University; Department of Pathology and Laboratory Medicine, Weill Cornell Medical College, New York, NY 10065, USA; The First Affiliated Hospital of Xiamen University-ICMRS Collaborating Center for Skeletal Stem Cells, State Key Laboratory of Cellular Stress Biology, Faculty of Medicine and Life Sciences, Xiamen; The First Affiliated Hospital of Xiamen University-ICMRS Collaborating Center for Skeletal Stem Cells, State Key Laboratory of Cellular Stress Biology, Faculty of Medicine and Life Sciences, Xiamen; Xiamen University; Department of Sports Medicine, Xiangya Hospital, Central South University, Changsha, 410000, China; Department of Sports Medicine, Xiangya Hospital, Central South University, Changsha, 410000, China; The First Affiliated Hospital of Xiamen University-ICMRS Collaborating Center for Skeletal Stem Cells, State Key Laboratory of Cellular Stress Biology, Faculty of Medicine and Life Sciences, Xiamen; The First Affiliated Hospital of Xiamen University-ICMRS Collaborating Center for Skeletal Stem Cells, State Key Laboratory of Cellular Stress Biology, Faculty of Medicine and Life Sciences, Xiamen; Xiamen University; Weill Cornell Medicine

**Keywords:** Osteogenesis imperfecta, Schnurri-3, Skeletal stem cell, Endothelial cell, Bone fracture

## Abstract

Osteogenesis imperfecta (OI) is a disorder of low bone mass and increased fracture risk due to a range of genetic variants that prominently include mutations in genes encoding type collagen. While it is well known that OI reflects defects in the activity of bone-forming osteoblasts, it is currently unclear whether OI also reflects defects in the many other cell types comprising bone, including defects in skeletal vascular endothelium or the skeletal stem cell populations that give rise to osteoblasts and whether correcting these broader defects could have therapeutic utility. Here, we find that numbers of skeletal stem cells (SSCs) and skeletal arterial endothelial cells (AECs) are augmented in *Col1a2*^*oim/oim*^ mice, a well-studied animal model of moderate to severe OI, suggesting that disruption of a vascular SSC niche is a feature of OI pathogenesis. Moreover, crossing *Col1a2*^*oim/oim*^ mice to mice lacking a negative regulator of skeletal angiogenesis and bone formation, Schnurri 3 (SHN3), not only corrected the SSC and AEC phenotypes but moreover robustly corrected the bone mass and spontaneous fracture phenotypes. As this finding suggested a strong therapeutic utility of SHN3 inhibition for the treatment of OI, a bone-targeting AAV was used to mediate *Shn3* knockdown, rescuing the *Col1a2*^*oim/oim*^ phenotype and providing therapeutic proof-of-concept for targeting SHN3 for the treatment of OI. Overall, this work both provides proof-of-concept for inhibition of the SHN3 pathway and more broadly addressing defects in the stem/osteoprogentior niche as is a strategy to treat OI.

## Introduction

Osteogenesis imperfecta (OI) is a disorder with heterogeneous genetic causes that prominently include mutations in the type I collagen genes, *COL1A1* and *COL1A2*. The hallmark of OI is low bone mass and skeletal fragility, resulting in susceptibility to fracture^1,2^. Currently, treatments including anti-resorptive drugs bisphosphonates (e.g. alendronate and zoledronate) are used for severe OI, though their efficacy in reducing fracture rates remains under investigation^3–11^. Bone anabolic agents, including are also under investigation for their potential benefit to OI patients in both preclinical and clinical trials^5,6^. Experimental therapies inhibiting TGF signaling are also under study for treatment of OI ^12^. Despite these advances and ongoing investigation there is still not a standard of care that has been clearly established to reduce fracture rates in OI and therefore a remaining unmet need for OI treatments. Whereas most therapeutic efforts in OI directly target well-established directly regulators of osteoblast or osteoclast activity, we hypothesized that skeletal microenvironmental dysregulation may also be a core feature of OI pathogenesis in addition to osteoblast intrinsic defects. Thus, targeting pathways that can both rescue these microenvironmental defects and cell intrinsic defects in osteoblast activity may provide a new and effective strategy for OI treatment.

Along these lines, it is increasingly appreciated that ancillary tissue types such as vascular endothelium present in bone actively contribute to osteogenesis^13,14^. Though it is known that deposition of collagen type I is critical for coupling between angiogenesis and osteogenesis during skeletal development, the degree to which the vascular microenvironment of bone is dysregulated in OI is unclear ^15,16^. It is also unknown how OI disrupts the early stem and progenitor cellular compartment in bone, especially a recently identified population of cells displaying formal evidence of stemness, SSCs that are defined through multicolor flow cytometry as lineage-Thy1-6C3-alpha-v integrin + CD200 + CD105- cells^17,18^.

In considering alternative approaches to augment bone formation and correct any potential vascular microenvironmental defects in OI, Schnurri-3 (SHN3, HIVEP3), a critical negative regulator of bone formation in both mice and humans offers an attractive novel approach ^19,20^. Mice lacking SHN3 display an osteosclerotic phenotype with profoundly augmented osteoblast activity leading to near absolute protection from age-related bone loss. In addition to the ability of SHN3 deficiency to directly drive the intrinsic bone formation activity of osteoblasts, SHN3 deficiency also acts in osteoblasts to control the skeletal vascular microenvironment by regulating production of a recently described osteoblast derived angiogenic factor, SLIT3^14,21^. Consistent with this, SHN3 deficient mice (*Shn3*^*−/−*^) mice display both enhanced bone formation and increased amounts of skeletal vascular endothelium, and both of these phenotypes are SLIT3 dependent^14^. Thus, the SHN3/SLIT3 signaling axis in osteoblasts offers a potential method to not only increase bone formation but also to address microenvironmental disruption occurring in skeletal disorders.

Here we sought to determine whether skeletal microenvironmental dysfunction occurs in OI and how this may impact early SSC populations, using a widely studied OI mouse model, *Col1a2*^*oim/oim*^ mice (OIM mice) displaying spontaneous fractures^22^. We further find evidence that correcting these defects in the skeletal vascular microenvironment can ameliorate the OI phenotype, as SHN3-deficiency can rescue the *Col1a2*^*oim/oim*^ model, correcting both the dysregulation in the SSC vascular niche and skeletal fragility in the *Col1a2*^*oim/oim*^ model.

## Results

### Altered skeletal vascular composition in Col1a2^oim/oim^ mice

**Figure 1:**
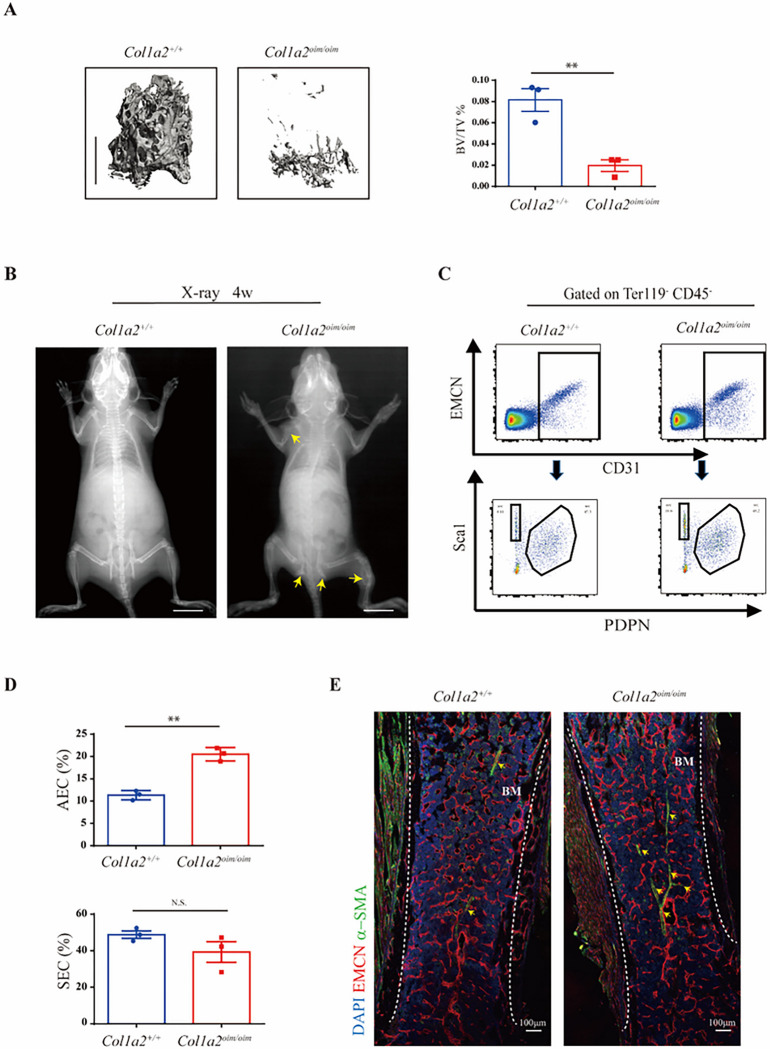
Altered skeletal vascular composition in *Col1a2*^*oim/oim*^ mice (A)Representative μCT images of trabecular bone in the distal femur (left) and bone volume/total volume (BV/TV) (right) in *Col1a2*^*+/+*^and *Col1a2* ^*oim/oim*^ male mice at 6 weeks of age. (n=3) (B) Representative X-ray images of *Col1a2*^*+/+*^and *Col1a2* ^*oim/oim*^ male mice at 4 weeks of age. Scale bars, 1cm, (n=3) (C)Representative flow cytometry plots and (D) quantitative analysis of arterial endothelial cells (AECs) and sinusoidal endothelial cells (SECs). Results are presented as mean ± s.e.m.; **P < 0.01 by an unpaired two-tailed Student’s t-test in all panels. (E)Representative confocal images (n = 3 total images per group) of femur sections from 3-week-old *Col1a2*^*+/+*^and *Col1a2* ^*oim/oim*^ male mice stained with EMCN (Red) and a-SMA (Green). Scale bars, 100 μm

Though deposition of collagen type I has been suggested to mediate coupling between angiogenesis and osteogenesis during bone formation, the degree to which disruption of this osteo-angio microenvironment contributes to OI and the specific forms of vascular endothelium that are impacted remain unclear ^14,23^. To address this, we selected *Col1a2*^*oim/oim*^ mice as a model for human OI. As anticipated, *Col1a2*^*oim/oim*^ mice showed a severe osteopenic phenotype and spontaneous bone fractures relative to littermate controls in 4 weeks old (Fig. 1A– B). To avoid potential confounding due to the presence of fracture healing responses, we selected adolescent *Col1a2*^*oim/oim*^ mice without spontaneous fractures for further examination.

Of note, a series of recent studies revealed that skeletal endothelial cells can be further divided into two separate subpopulations, arterial endothelial cells (AECs) and sinusoidal endothelial cells (SECs) and each of which play a distinct role in supporting osteogenesis. We then established a new multi-color flow cytometry using the combination of podoplanin (PDPN) and Sca-1 to distinguish AECs from SECs in the marrow cavity (Fig. 1C). Based on this, the population of AECs but not SECs was significantly elevated in bones of *Col1a2*^*oim/oim*^ mice (Fig. 1D). Immunofluorescence analysis confirmed that the abundance of arterial vessels was indeed increased substantially in *Col1a2*^*oim/oim*^ mice (Fig. 1E). Of note, this effect was specific for AEC, as other forms of skeletal endothelium, including CD31^hi^ endomucin^hi^ (EMCN^hi^) endothelial cells^23^, where not altered (Fig. S1A-B). Thus, expansion of AEC is a feature of OI models.

### Elevated abundance of skeletal stem cells in Col1a2^oim/oim^ mice

**Figure 2:**
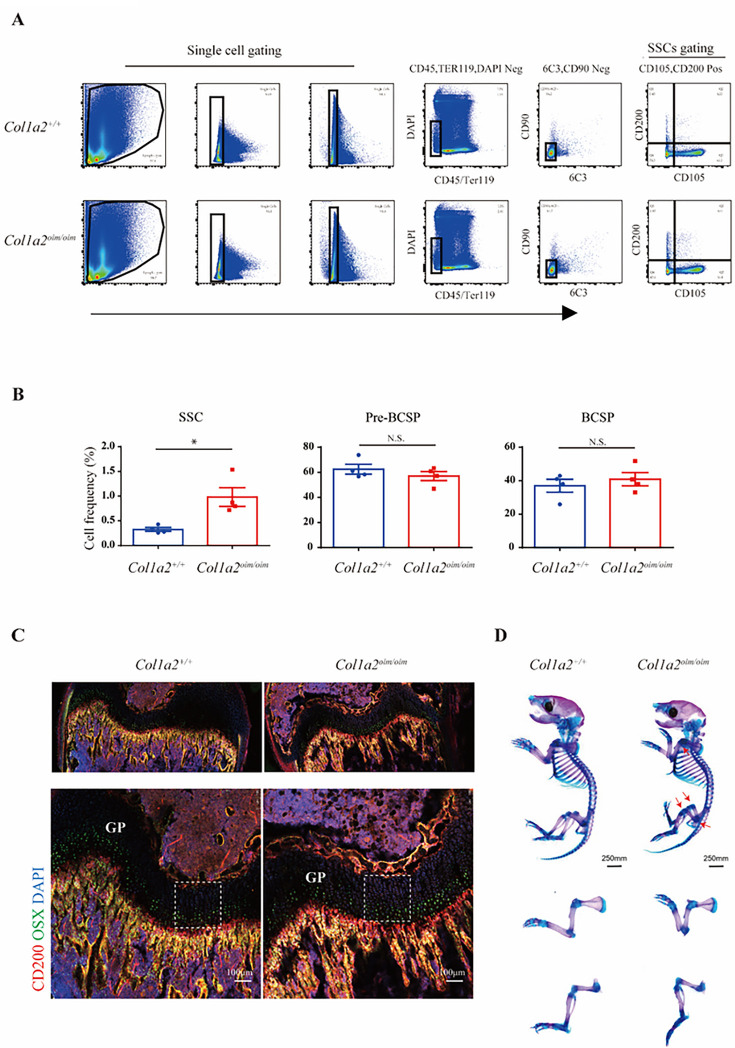
Elevated abundance of skeletal stem cells in *Col1a2*^*oim/oim*^ mice (A) Flow cytometry gating strategies for analysis of SSC, pre-BCSP and BCSP proportions. (B)Dot plot for analysis of SSC, pre-BCSP and BCSP proportions in *Col1a2*^*oim/oim*^ mice and *Col1a2*^*+/+*^ littermate controls. Results represented as mean ± s.e.m.; *P < 0.05 by an unpaired two-tailed Student’s t-test in all panels. (C)Representative confocal images (n = 3 total images per group) of femur sections from 3-week-old *Col1a2*^*+/+*^and *Col1a2* ^*oim/oim*^ male mice stained with CD200 (red) OSX (green) and DAPI (blue). The growth plate is marked with a dashed line. (Top, lower power; Bottom, higher power). Scale bars, 250 μm (low power) and 100μm (high power). (D)Alcain blue and alizarin red staining of skeletal preparations of newborn mice at postnatal day 3. Scale bars, 250 mm

Skeletal stem cells (SSCs) serve as the ultimate source of all bone forming osteoblasts, therefore perturbations in the SSC compartment are likely central to many skeletal disorders^24,25^. Considering that AECs regulate hematopoietic stem cell (HSC) proliferation^26^, we then considered whether the AEC expansion seen in OI may similarly translate to SSC alterations. To evaluate this, we utilized an established multi-color flow cytometry panel identifying SSCs^18^ to analyze skeletal stem/progenitor cells populations in long bones of *Col1a2*^*oim/oim*^ mice (Fig. 2A). Interestingly, the abundance of immunophenotypic SSCs was elevated in *Col1a2*^*oim/oim*^ mice, yet the amounts of pre-bone cartilage skeletal progenitors (pre-BCSPs) and bone cartilage skeletal progenitors (BCSPs), SSC-derived non-stem progenitors^17^, was unchanged (Fig. 2B). This, alteration in the ratio of different stages of SSC maturation indicates that OI impacts the stem cell differentiation hierarchy. Consistent with this, the number of CD200 positive cells was obviously increased in the primary spongiosum near the growth plate in *Col1a2*^*oim/oim*^ mice, a region characterized as housing more SSCs and more artery/arterioles^16,17,27,28^ (Fig. 2C). Moreover, delayed osteogenesis attributed to impaired mineralization was observed in *Col1a2*^*oim/oim*^ mice evidenced by the whole-mount skeletal staining in neonatal mice (Fig. 2D), with the early emergence of this phenotype being consistent with alterations in the early SSC compartment. Given increase in both SSCs and AECs seen in *Col1a2*^*oim/oim*^ mice, disruptions in an angiogenic SSC niche are likely contributors to the overall OI skeletal phenotype.

### Col1a2 ^oim/oim^ SSCs lacking osteogenic capacity are highly proliferative

**Figure 3:**
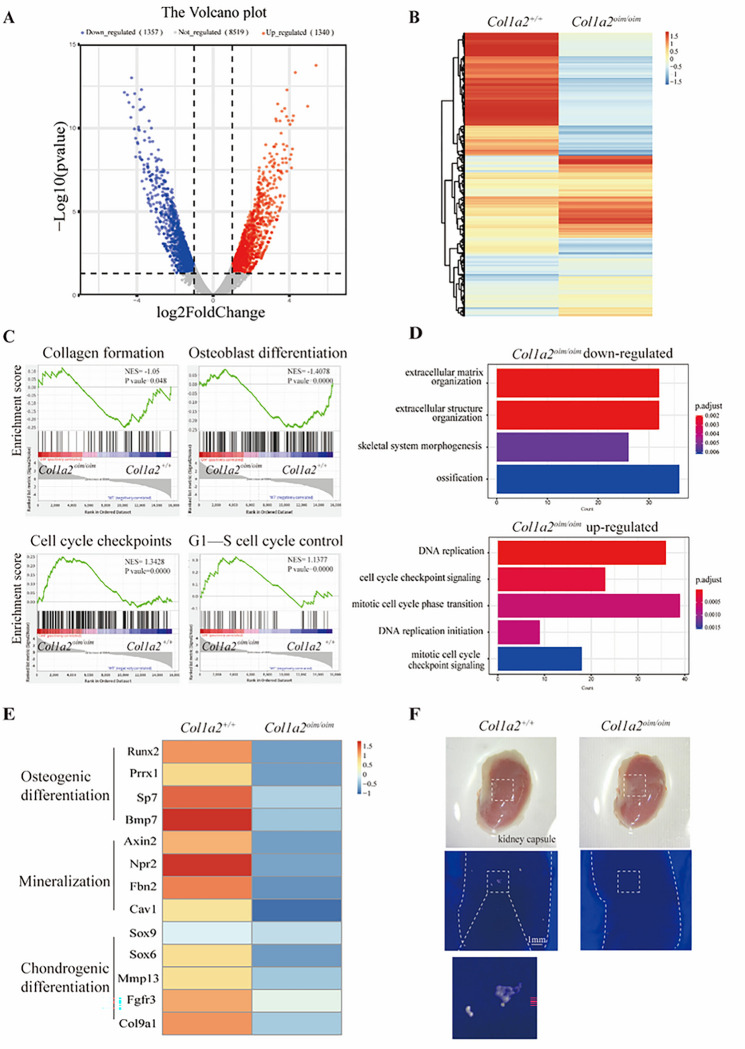
*Col1a2* ^*oim/oim*^ SSCs display cell intrinsic transcriptional alterations and functional defects in osteogenic capacity (A) Volcano plot illustrating differentially regulated gene expression from Bulk RNA-seq analysis between the 7-day postnatal control *Col1a2*^*+/+*^ mice SSCs and SSCs from the *Col1a2*^*oim/oim*^ mice. (B) Hierarchical clustering based on Euclidian distance using Illumina TruSeq RNA Sample Preparation kit and sequenced on Illumina HiSeq 4000. Blue, downregulated; Red, upregulated. (C) Gene set enrichment plot demonstrated activation of Collagen formation, Osteoblast differentiation, Cell cycle checkpoints and G1-S cell cycle control signaling. *Col1a2*^*+/+*^ and *Col1a2*^*oim/oim*^ represent mixture 3 biologically distinct samples. The expression pattern of genes involved in the Collagen formation, Osteoblast differentiation, cell cycle checkpoints and G1-S cell cycle control signaling set in the analysis database is shown. NES, normalized enrichment score. Blue, downregulated; Red, upregulated. (D) Gene Ontology (GO) functional clustering of the interested downregulated and upregulated biological process (BP) in *Col1a2*^*oim/oim*^ mice SSCs. (Top, downregulated; Bottom, upregulated) (E) Heatmap for osteogenic cartilage-related gene expression in *Col1a2*^*+/+*^mice SSCs (left) and *Col1a2*^*oim/oim*^ mice SSCs (right). (F) General view (top) and microCT results (middle and bottom) of in vivo organoid formation assay through kidney capsule transplantation. ~2000 SSCs isolated from 7-day postnatal *Col1a2*^*+/+*^ and *Col1a2*^*oim/oim*^ mice were embedded with Matrigel and implanted in kidney capsule for 4 weeks. Area containing transplanted cells is shown by the dashed white line (general view) and red line (microCT), respectively.

To gain mechanistic insight into how SSCs were influenced in *Col1a2*^*oim/oim*^ mice, we performed RNA-sequencing (RNA-seq) transcriptional profiling of SSCs derived from neonatal *Col1a2*^*oim/oim*^ mice and littermate gender-matched WT controls. SSCs from *Col1a2*^*oim/oim*^ mice exhibited distinctive transcriptomic features including 1357 down-regulated genes and 1340 up-regulated genes compared with SSCs from WT controls (Fig. 3A–B). By gene set enrichment analysis (GSEA), *Col1a2*^*oim/oim*^ SSCs displayed transcriptional features of impaired collagen synthesis capacity and impaired osteoblastogenesis, consistent to the delayed mineralization and skeletal phenotypes in *Col1a2*^*oim/oim*^ mice (Fig. 3C–D, upper). *Col1a2*^*oim/oim*^ SSCs also displayed transcriptional features of an increased mitotic cell cycle (Fig. 3C–D, lower), corresponding to the elevated abundance of SSCs in *Col1a2*^*oim/oim*^ mice (Fig. 2A–B). Moreover, expression of genes related to stemness and early osteogenesis and endochondral ossification were also decreased in SSCs derived from *Col1a2*^*oim/oim*^ mice (Fig. 3E). To determine if these transcriptional alterations in SSCs translate into cell-intrinsic functional differences, we sorted and transplanted the equal amounts of SSCs into kidney capsule for an organoid bone formation assay. μCT analysis showed that *Col1a2*^*oim/oim*^ SSCs display functional defects in mineralization (Fig. 3F). Taken together, these findings implicate a broader set of cellular pathology, including defects in SSCs and AECs, beyond functional defects in mature osteoblasts in OI.

### Deletion of SHN3 improves bone properties in Col1a2^oim/oim^ mice

**Figure 4:**
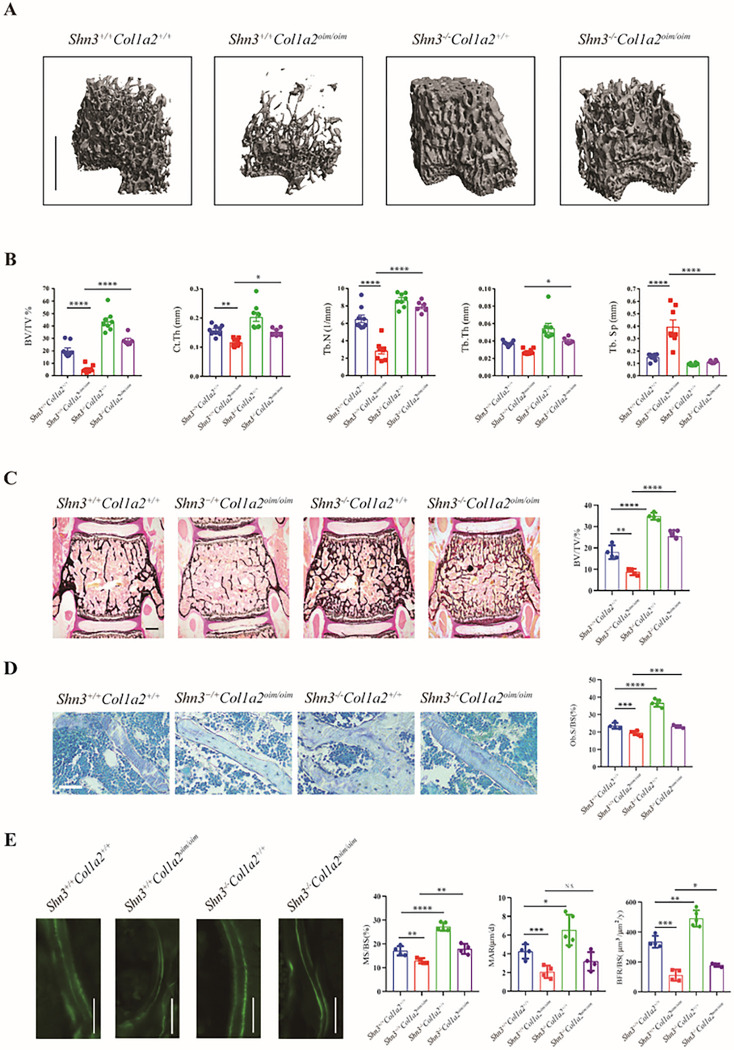
Deletion of SHN3 improves bone properties in *Col1a2*^*oim/oim*^ mice (A) Representative μCT images of the trabecular bone in the distal femur metaphysis and (B) relative quantitative analysis of BV/TV in *Shn3*^*+/+*^*Col1a2*^*+/+*^and *Shn3*^*+/+*^*Col1a2* ^*oim/oim*^ and *Shn3*^*−/−*^*Col1a2*^*+/+*^ and *Shn3*^*−/−*^*Col1a2* ^*oim/oim*^ male mice. Analysis at 6 weeks of age. Scale bars, 1 mm. Values were presented as mean ± s.e.m.; *P < 0.05, **P < 0.01 and ****P < 0.0001 by an unpaired two-tailed Student’s t-test in all panels. (C) Representative images and BV/TV analysis of Von Kossa staining and the quantification ofhistomorphometric parameters of L3 vertebrae in *Shn3*^*+/+*^*Col1a2*^*+/+*^and *Shn3*^*+/+*^*Col1a2*^*oim/oim*^ and *Shn3*^*−/−*^ *Col1a2*^*+/+*^ and *Shn3*^*−/−*^*Col1a2* ^*oim/oim*^ male mice at 6 weeks of age, Scale bars, 500 μm. (n=4 for each group) (D) Representative histological images of the L3 vertebrae with Toluidine blue staining and the quantification of histomorphometric parameters in *Shn3*^*+/+*^*Col1a2*^*+/+*^and *Shn3*^*+/+*^*Col1a2* ^*oim/oim*^ and *Shn3*^*−/−*^*Col1a2*^*+/+*^ and *Shn3*^*−/−*^*Col1a2* ^*oim/oim*^ male mice at 6 weeks of age. Trabecular osteoblast surface/bone surface (Ob.S/BS) (%) are shown. Scale bars, 50μm. (*Shn3*^*+/+*^*Col1a2*^*+/+*^, n=4, *Shn3*^*+/+*^*Col1a2* ^*oim/oim*^ ,n=5, *Shn3*^*−/−*^*Col1a2*^*+/+*^,n=5, *Shn3*^*−/−*^*Col1a2* ^*oim/oim*^,n=4) (E) Representative images of calcein double labeling and quantification of histomorphometric parameters of the L3 vertebrae in *Shn3*^*+/+*^*Col1a2*^*+/+*^and *Shn3*^*+/+*^*Col1a2* ^*oim/oim*^ and *Shn3*^*−/−*^*Col1a2*^*+/+*^ and *Shn3*^*−/−*^*Col1a2* ^*oim/oim*^ male mice at 6 weeks of age. Trabecular mineralizing surface/bone surface (MS/BS)(%), mineral apposition rate (MAR, μm day^−1^), bone formation rate/bone surface.(BFR/BS) (μm^3^μm^−2^yr^−1^) are shown. Scale bars, 100μm. (*Shn3*^*+/+*^*Col1a2*^*+/+*^*,* n=3, *Shn3*^*+/+*^*Col1a2* ^*oim/oim*^ , n=4, *Shn3*^*−/−*^*Col1a2*^*+/+*^*,*n=4, *Shn3*^*−/−*^*Col1a2* ^*oim/oim*^, n=4)

There remains a substantial unmet clinical need for OI treatments that reduce fracture risk in OI^11,29^. We have previously reported that SHN3 acts as a cell intrinsic negative regulator of both osteoblast bone formation activity and the ability of osteoblasts to promote an osteoanabolic vascular microenvironment in bone^14^. To evaluate both whether SHN3 inhibition is a potential therapeutic approach to treat OI and also whether SHN3-mediated regulation of the skeletal vascular microenvironment is relevant to OI phenotypes, we intercrossed *Shn3*^*−/−*^ mice with *Col1a2*^*oim/oim*^ mice ^14^. Ablation of SHN3^19,30^ provided a complete or near complete reversal of the low bone mass observed in both trabecular and cortical bone compartments in *Col1a2*^*oim/oim*^ mice (Fig. 4A, B). Histomorphometric analysis revealed that SHN3-deficiency was sufficient to reverse the osteopenic phenotype and attenuated osteoblast numbers in *Col1a2*^*oim/oim*^ mice (Fig. 4C, D). Likewise, the decrease in bone formation rate (Fig. 4E) observed in *Col1a2*^*oim/oim*^ mice was normalized through additional deletion of *Shn3* (Fig. 4F, G). Thus, deletion of *Shn3* is capable to block OI-induced bone loss by normalizing bone remodeling.

### Ablation of Shn3 prevents spontaneous fractures in Col1a2^oim/oim^ mice

**Figure 5:**
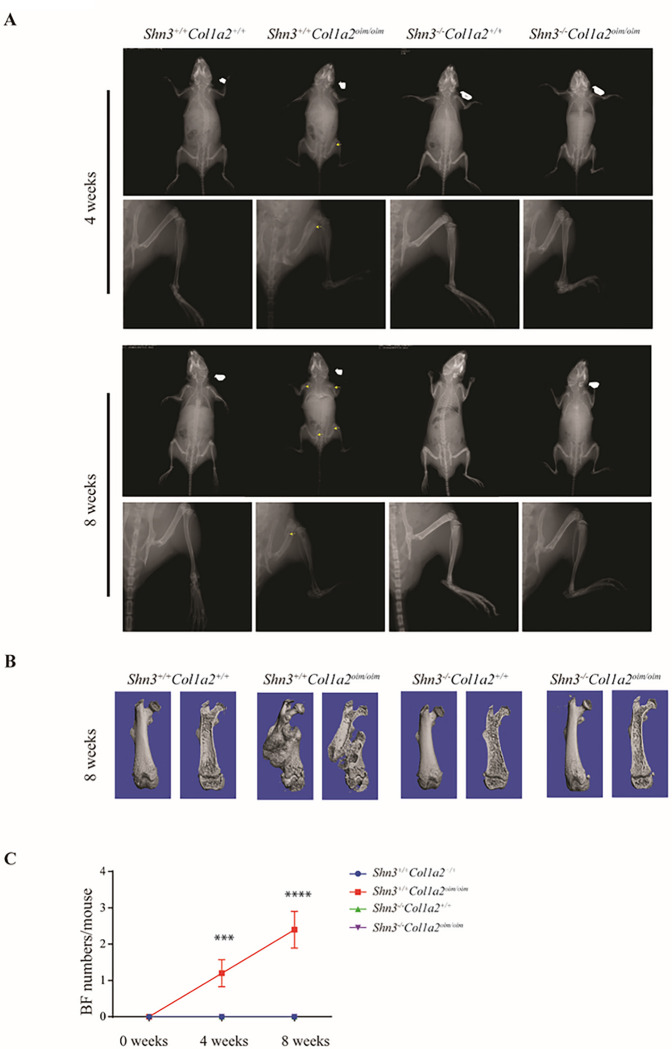
Ablation of *Shn3* prevents spontaneous fractures in *Col1a2* ^*oim/oim*^ mice (A) Representative X-ray image of *Shn3*^*+/+*^*Col1a2*^*+/+*^and *Shn3*^*+/+*^*Col1a2* ^*oim/oim*^ and *Shn3*^*−/−*^*Col1a2*^*+/+*^ and *Shn3*^*−/−*^*Col1a2* ^*oim/oim*^ male mice at 4week and 8 weeks age. Scale bar: 2 mm. (B) Reconstruction of μCT data reflected spontaneous bone fracture in *Shn3*^*+/+*^*Col1a2*^*+/+*^ and *Shn3*^*+/+*^*Col1a2* ^*oim/oim*^ and *Shn3*^*−/−*^*Col1a2*^*+/+*^ and *Shn3*^*−/−*^*Col1a2* ^*oim/oim*^ male mice at 8 weeks age. (C) Relative quantification of spontaneous bone fracture numbers in *Shn3*^*+/+*^*Col1a2*^*+/+*^ and *Shn3*^*+/+*^*Col1a2* ^*oim/oim*^ and *Shn3*^*−/−*^*Col1a2*^*+/+*^ and *Shn3*^*−/−*^*Col1a2* ^*oim/oim*^ male mice after the birth of 1 weeks. Analysis at 1 week, 5weeks and 9 weeks of age. Values represent mean ± s.e.m.; **P < 0.01 and ***P < 0.001 by an unpaired two-tailed Student’s t-test in all panels.

While it is encouraging that SHN3-deficient *Col1a2*^*oim/oim*^ mice display a restoration of bone formation parameters, the most clinically relevant endpoint is the prevention of fractures, the major source of morbidity in clinical OI. Indeed, *Col1a2*^*oim/oim*^ mice displayed spontaneous fractures starting at 3 weeks of age that increased in frequency until an average of 3 fractures per mouse could been seen at 8 weeks of age (Fig. 5A–C). Strikingly, we found SHN3 deficiency is able to completely prevent the spontaneous bone fractures in *Col1a2*^*oim/oim*^ mice as no fractures occurred in *Shn3*^*−/−*^*Col1a2*^*oim/oim*^ mice. This protection from fracture in *Shn3*^*−/−*^*Col1a2*^*oim/oim*^ mice also correlated with rescue of the running seen in *Col1a2*^*oim/oim*^ mice, perhaps reflecting the effect of fracture-associated stress on skeletal growth. Thus, SHN3 deficiency not only normalizes bone formation in *Col1a2*^*oim/oim*^ mice but completely prevents the signature spontaneous fractures occurring in this OI model.

### SHN3 deficiency corrects the vascular and SSC compositional changes in Col1a2^oim/oim^ mice

**Figure 6:**
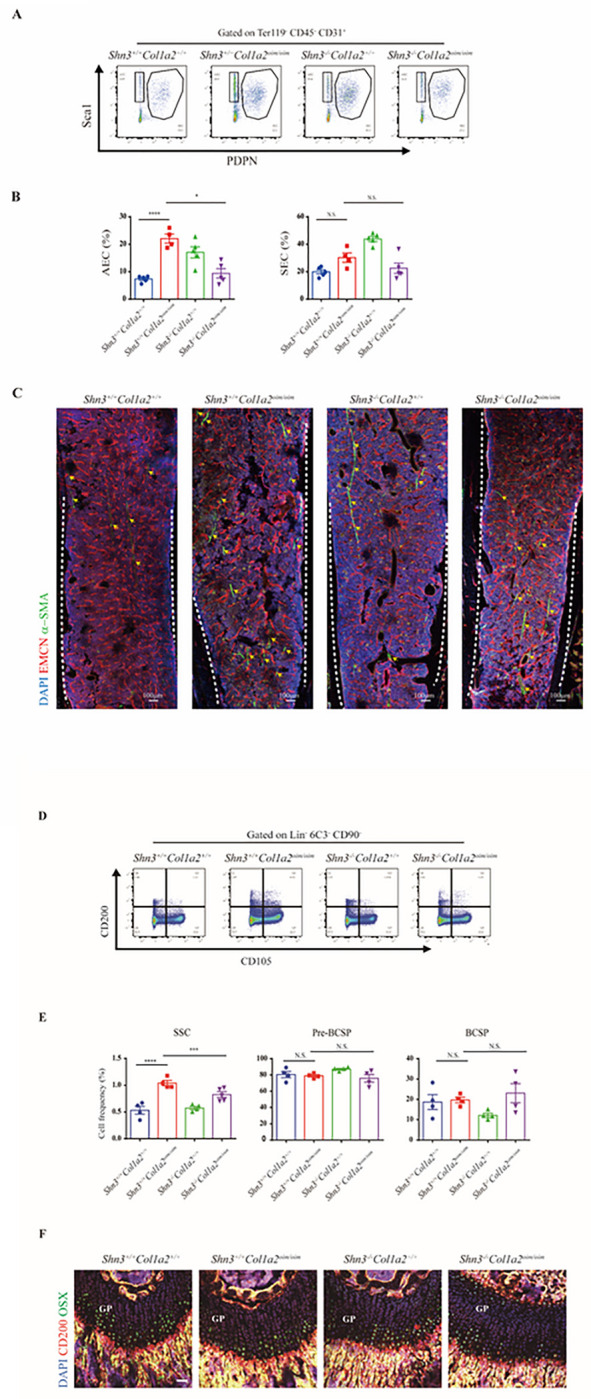
SHN3 deficiency corrects abnormal vascular and SSC composition in *Col1a2* ^*oim/oim*^ mice (A) Experimental strategy and representative flow cytometry plots (B) and relative frequency of arterial endothelial cells (AECs) and sinusoidal endothelial cells (SECs) (C) Representative confocal images (n = 3 total images per group) of femur sections from 3-week-old *Shn3*^*+/+*^*Col1a2*^*+/+*^ and *Shn3*^*+/+*^*Col1a2* ^*oim/oim*^ and *Shn3*^*−/−*^*Col1a2*^*+/+*^ and *Shn3*^*−/−*^*Col1a2* ^*oim/oim*^ male mice stained with EMCN (Red) and a-SMA (Green), DAPI (Blue). Scale bars, 100 μm. (D) Experimental strategy and representative flow cytometry plots (B) and relative frequency of SSCs, Pre-BCSP, BCSP. (E) Representative confocal images (n = 3 total images per group) of femur sections from 3-week- *Shn3*^*+/+*^*Col1a2*^*+/+*^ and *Shn3*^*+/+*^*Col1a2* ^*oim/oim*^ and *Shn3*^*−/−*^*Col1a2*^*+/+*^ and *Shn3*^*−/−*^*Col1a2* ^*oim/oim*^ male mice stained with antibodies recognizing CD200 (Red) and OSX (Green) or DAPI(Blue). Scale bars, 50 μm

We next evaluated whether the ability of SHN3-deficiency to rescue the spontaneous fractures in *Col1a2*^*oim/oim*^ mice reflected a correction of the skeletal microenvironment. To this end, we analyzed the cellular composition of the vascular endothelium and SSC compartments in *Shn3*^*−/−*^ *Col1a2*^*oim/oim*^ mice using a previously reported multi-color flow cytometry panel^17^. Strikingly, *Shn3* deficiency rescued the skeletal vascular pathology in *Col1a2*^*oim/oim*^ mice. *Shn3*-depletion attenuated the AEC expansion seen in *Col1a2*^*oim/oim*^ mice while not impacting the amount of SECs (Fig. 6A–B). Interestingly, SHN3 deficiency alone did not notably alter the amount of SSCs or their production of downstream cell types. Despite this, *Shn3*-deficiency reversed the SSC expansion seen in *Col1a2*^*oim/oim*^ mice, limiting the hyper proliferative phenotype seen in the SSC-enriched primary spongiosum region in *Col1a2*^*oim/oim*^ mice adjacent to the growth plate (Fig. 6C–E). These results implied that the expansion of SSCs is closely linked to the AECs expansion seen in *Col1a2*^*oim/oim*^ mice, in line with emerging evidence that endothelial cells play a critical role in the SSC niche^31^. Thus, deletion of SHN3 corrected the cellular alterations in AECs and SSCs seen in *Col1a2*^*oim/oim*^ mice, correcting the cellular perturbations in seen with this OI model (Fig. 6A, C). This finding also links correction of these cellular pathologies with overall rescue of the OI phenotype.

### Shn3 -silencing is a candidate therapeutic approach for OI

**Figure 7:**
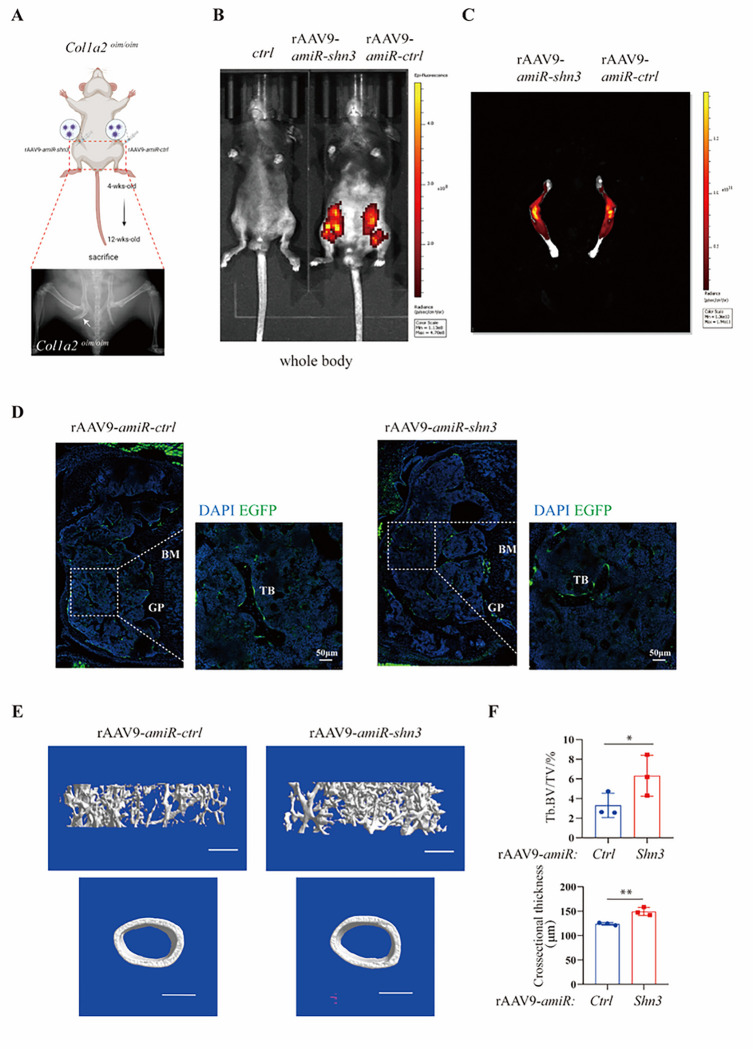
SHN3-silencing is a candidate therapeutic approach for OI (A) Schematic diagram of strategy for rAAV9-*amiR-ctrl* or *amiR-shn3* injected into knee joints in 4 weeks old *Col1a2*^*oim/oim*^, rAAV9-*amiR-ctrl* in the left hindlimbs and *amiR-shn3* in the right hindlimbs, sacrificed in 12 weeks old. (B)(C) Two months after i.a. injection of rAAV9 carrying amiR-ctrl or amiR-shn3 into knee joints of 1month-old male mice, EGFP expression was assessed by IVIS Lumina III optical imaging. </p/> (D) Two months after i.a. injection of rAAV9 carrying *amiR-ctrl* or *amiR-shn3* into knee joints of 1-month-old male mice, the vector-driven EGFP signal in trabecular bone was visualized by fluorescence microscopy of cryo-sectioned femurs. Scale bars, 50μm (E) Two months after i.a. injection of rAAV9 carrying *amiR-ctrl* or *amiR-shn3* into knee joints of 1-month-old male mice, femoral trabecular bone mass was assessed by μCT. (F) Representative 3D reconstruction and relative quantification are displayed (n = 3 per group) Scale bars, 100μm

AAV-based gene therapy is emerging as an attractive modality for the treatment of skeletal disorders due to its ability to potentially mediate long-lasting effects after a single treatment and the ability to address therapeutic targets that are challenging for traditional small molecule or biologic therapies, such as *Shn3*^32–34^. Previous studies utilized AAV serotype-9 to deliver a *Shn3*-silencing construct that mediated a robust reduction in *Shn3* expression in osteoblastic lineage cells and accordingly augmented bone mass under both baseline physiologic conditions and in a mouse model of post-menopausal osteoporosis.^35^ To investigate whether a gene therapy approach to inhibit SHN3 expression is capable of treating OI, we constructed replication-defective recombinant AAV serotype 9 (rAAV9) harboring enhanced green fluorescent protein (EGFP)-expressing plasmids that additionally bear either a *Shn3* targeting artificial microRNA (rAAV9-amiR-Shn3) or a miRNA-control (rAAV9-amiR-Ctrl) as previously described^35^. This vector displayed robust tropism of for osteoblasts and favorable relative specificity of payload delivery after intraarticular injection in prior studies^35^. We administered rAAV9-amiR-Shn3 or rAAV9-amiR-Ctrl to *Col1a2*^*oim/oim*^ mice via intraarticular injection at 4 weeks of age into contralateral limbs of the same mouse and evaluated their skeletal phenotype at 12 weeks of age (Fig. 7A). Through IVIS optical imaging, expression of eGFP was predominantly localized to the hindlimb of rAAV9-injected mice, with both the femur and tibia displaying high intensity eGFP expression (Fig. 7B–C). Fluorescence microscopy confirmed that bone-lining osteoblasts were effectively transduced by the rAAV9 vector (Fig. 7D). Furthermore, trabecular bone volume was significantly higher in rAAV9-amiR-Shn3 administered limbs compared to contralateral rAAV9-amiR-Ctrl limbs and cortical bone is also significantly thickened, which reduces the probability of fractures (Fig. 7E–F). This provides proof-of-concept that postnatal therapeutic targeting of *Shn3* is able to reverse the osteopenia seen in the *Col1a2*^*oim/oim*^ mice and provides specific demonstration of an AAV-based gene therapy approach to *Shn3* targeting.

## Discussion

Currently, there remains a substantial unmet clinical need for methods to treat OI, as it whether anti-resorptive drugs such as bisphosphonates or other repurposed osteoporosis therapies will correct the increased fracture rate that is the signature clinical issue of OI remains an area of active investigation. In part this unmet need is a call for both an improved mechanistic understanding of OI and for investigation into innovative means to correct or compensate for the cellular, architectural and bone materials properties deficits driving skeletal fragility in OI.

Recent advances in single cell transcriptome analysis and the FACS-based definition of skeletal cell types have created new avenues for understanding the cellular pathogenesis of OI and other skeletal disorders, by allowing the first molecular definitions of the skeletal and endothelial cell populations comprising bone. Here we have made one of the first applications of these approaches to define alterations in skeletal cell types to a specific skeletal disease^36^, finding that the amount of SSCs and skeletal AECs are both significantly elevated in bones of *Col1a2*^*oim/oim*^ mice. Moreover, correction of the OI phenotype leads to a reversal this expansion in SSCs and AECs, arguing that this expansion is linked to OI pathogenesis. While it was well established that functional defects in mature osteoblasts are central to the pathogenesis of OI, it was unclear if there was a broader dysfunction in SSCs^37^. Here, we first found that the amount of SSC was elevated in the *Col1a2*^*oim/oim*^ OI model, implicating altered stem cell dynamics in OI.

The functional and physical coupling between osteogenesis and angiogenesis is increasingly emerging as a critical point of dysfunction in skeletal disorders and also a promising but largely unexplored therapeutic opportunity. Previously, we found that SHN3 acts in osteoblasts to regulate production of SLIT3, which in turn acts as a skeletal specific angiogenic factor^14^. Through this angiogenic activity, SLIT3 in turn creates a skeletal vascular microenvironment that enables anabolic bone formation. We here find a linkage between AEC abundance and SSC expansion in OI that in turn suggests a broader functional linkage between AECs and SSCs, perhaps with AECs serving as part of the SSC niche. This fits with an emerging picture that specific skeletal progenitor populations may be specifically localized to perivascular or peri-arteriolar regions, and provides some of the first direct evidence that vascular modulation produces corresponding changes in SSCs as functional evidence for a perivascular SSC niche ^31,38,39^. This also raises the question of whether SLIT3 is a specific regulator of SSC abundance through a selective ability to modulate AECs as opposed to SECs, suggesting a model whereby the composition of skeletal vascular endothelium can be tuned by a series of subset vessel-type specific angiogenic factors, possibly including PDGF-bb, VEGF isoforms or others, that in turn govern the composition of the pool of early skeletal progenitors^40^. In this manner, a vascular targeted osteoanabolic agent may be synergistic with a traditional osteoblast targeted osteoanabolic through activity to “prime” the osteoanabolic effect both by preparing the pool of early vasculature associated stem and progenitor cells and also by creating a microenvironment that favors bone formation. Thus, the SHN3-targeting gene therapy approach taken here is also anticipated to be complimentary to either established osteoanabolic or antiresorptive therapeutics or to emerging therapeutic approaches that specifically target the molecular pathogenesis of OI, such as anti TGF-β antibodies^5,12^.

Whether achieved via a gene therapy-based approach as tested here or via other methods, inhibition of SHN3 is an attractive approach for treating OI. Prior mechanistic studies have shown that, in addition to the above ability of SHN3 to regulate the bone vascular microenvironment through regulation of SLIT3 secretion, SHN3 mediates a cell intrinsic effect in osteoblasts to suppress bone formation. This occurs in part via the ability of SHN3 to suppress ERK-mediated phosphorylation of selected substrates^19,30^. While SHN3 is broadly expressed, the phenotypes associated with SHN3 deficiency appear to be limited to the skeleton, which is promising for the possibility that SHN3-targeted therapies will display a favorable effect to toxicity profile.

In summary, this project has identified new cellular features of OI in the SSC and vascular compartments of bone and identified preclinical evidence supporting a new therapeutic approach centering on inhibition of the SHN3 pathway with an AAV delivered payload. We anticipate that this will not only motivate further development of AAV and SHN3-based gene therapeutic approaches, but moreover provide evidence for marked disruption in the vascular and SSC compartments as a feature of OI that could be central to disease pathogenesis.

## Materials and Methods

### Animals

*Col1a2*^*oim/oim*^ mice were obtained from the Jaxson Laboratory (B6C3Fe *a/a-Col1a2*^*oim*^/J, Stock No: 001815, Bar Harbor, ME, USA); *Shn3*^*−/−*^ mice were described in our previous studies^14,19^. Dual heterozygous *Shn3*^*+/−*^*Col1a2*^*oim/+*^ mice were used for breeding to generate *Shn3*^*+/+*^*Col1a2*^*+/+*^ mice, *Shn3*^*+/+*^*Col1a2*^*oim/oim*^ mice, *Shn3*^*−/−*^*Col1a2*^*+/+*^ mice and *Shn3*^*−/−*^*Col1a2*^*oim/oim*^ mice. All mice were housed up to four per cage under a 12-hour light-dark cycle with chow ad libitum in the Laboratory Animal Center at the Xiamen University. All mouse experiments were handled according to the protocols approved by the Institutional Animal Care and Use Committee of Xiamen University Laboratory Animal Center.

### Radiography and micro-CT analysis

Whole-body radiographs of experimental mice were captured by a Faxitron X-ray system. We defined fractures in the humerus, forearms, femurs and tibias by bone deformity and callus formation. We performed μCT scanning using a μCT 35 system (Scanco Medical, Sweden) at the Weill Cornell-Citigroup Biomedical Imaging Core according to the parameters in our previous study^21^. The analysis was conducted by a technician blinded to the genotypes of the mice under analysis.

### Histology and dynamic histomorphometry

We injected the experimental and control mice intraperitoneally with a dose of 20mg/kg calcein on days 1 and 5 before sacrifice for measurement of bone formation rate. Resin embedding and sectioning without decalcification was performed for von Kossa staining, toluidine blue staining and TRAP staining as described in our previous study^41^. We then use the Osteomeasure System (OsteoMetrics, Atlanta, USA) for histomorphometric analysis as previously described^13^.

### Immunofluorescence staining

Frozen sectioning for skeletal immunofluorescence staining were prepared according to a published protocol and our recent studies^14,18,42^. Primary antibodies (rat anti-mouse CD200 (Abcam), rabbit anti-mouse SP7 (Abcam, USA), goat anti-mouse CD31 (R&D, USA) and rat anti-mouse endomucin (Santa Cruz, USA)) and species-specific secondary antibodies with Alexa Fluor 488 and 594 (Invitrogen, USA) were used in this study.

### Flow cytometry analysis and cell sorting

To analyze skeletal endothelial cells, 3μg PE-anti-mouse PDPN (Biolegend, USA) were injected intravenously. After 10 min, femurs were isolated from mutant mice and littermate controls and were crushed in Hank’s balanced salt solution with 10 mmol·L^− 1^ HEPES. For the enzymatic digestion, 2.5 mg·mL^− 1^ Collagenase A and 1 U·mL^− 1^ Dispase II were added for a 15 min incubation at 37°C with gentle agitation. Next, PBS containing 2% FBS and 2 mmol·L^− 1^ EDTA were added to stop the digestion, and the resulting suspension was filtered through a 70 μm cell strainer and washed twice with PBS. After blocking nonspecific staining with an anti-mouse CD16/CD32 antibody (BD Biosciences, USA) for 15 min on ice, the cells were stained with Alexa Fluor 700-conjugated Ter119 antibody (BD), Alexa Fluor 700-conjugated CD45 antibody (BD Biosciences, USA), FITC-conjugated CD31 (BioLegend, USA) and APC-conjugated EMCN antibody (eBioscience, USA). For skeletal stem cell analysis, the cells were stained with a FITC-conjugated CD45 antibody (BioLegend, USA), APC/Cy7-conjugated Ter119 antibody (BioLegend, USA), PE-conjugated CD31 antibody (eBioscience, USA), PerCP-Cy5.5-conjugated 6C3 antibody (BioLegend, USA), BV605-conjugated CD90.2 antibody (BioLegend, USA) BV421-conjugated CD200 antibody (BD Biosciences, USA) and PE/Cy7-conjugated CD105 antibody (BioLegend, USA) for 30 min on ice. 1 μg·mL^−1^ DAPI solution (BD Biosciences, USA) was used for live/dead exclusion with combination of FSC and SSC. Cell sorting was performed with FACS Aria II (BD, San Jose, CA, USA) and analyzed using FlowJo software (Tree Star, Ashland, OR, USA).

### Kidney capsule transplantation model

Kidney capsule transplantation models were performed as previously described^17,18^. 2-month-old male mice were anesthetized with isoflurane and shaved on the right flank and lower back prior to surgical site sterilization. A 5-mm incision was made below the rib arch to expose the right kidney. And a 2-mm pocket was opened in the renal capsule to implant a 5–8uL Matrigel plug (Corning, USA) containing 2000 to 3000 SSCs. After implantation, the renal capsule was then sealed with an electrocautery. The kidney was then relocated back to the peritoneal cavity prior to layered Vicryl suture (Johnson & Johnson, USA) closure of the peritoneal and skin incisions. Recipients were euthanized by CO_2_ narcosis 4 weeks after transplantation. Kidneys were carefully dissected, fixed with 4% paraformaldehyde (PFA) for 6 hours and mineralized organoids were assessed by μCT analysis.

### Bulk RNA sequencing

Bulk RNA sequencing was performed on SSCs isolated from 7-day postnatal *Col1a2*^*oim/oim*^ mice and littermate gender-matched controls as previously described^18^. In brief, cDNA libraries were generated with the Illumina TruSeq RNA Sample Preparation kit and sequenced on an Illumina HiSeq 4000. HISAT2(v2.2.1) was used to align raw sequencing reads to the mm10 mouse reference genome. Differential expression analysis was performed using edgeR package (v 3.36.0, Bioconductor). Genes with expression level lower than 1 CPM (counts per million) were considered as displaying low expression and were excluded. The heatmap and volcano plot were generated to visualize differential genes utilizing pheatmap(v1.0.12) and ggplot2 package(v3.3.6), respectively. For gene functional annotation analysis, GO and pathway enrichment analysis was performed for up-regulated/down-regulated DEGs using clusterProfiler package(v4.4.4), and the P values of enriched terms were adjusted by Benjamini-Hochberg method and terms were filtered by setting pvalueCutoff to 0.05. Gene set enrichment analysis was performed using the GSEA software(v4.3.2) for linux. We selected gene sets of ontology and curated signaling pathways from the MSigDB Database(v2022.1, https://www.gsea-msigdb.org) to identify differential gene ontology and pathways enriched in *Col1a2*^*oim/oim*^ and littermate mice sample subsets.

### rAAV9-mediated silencing of Shn3, intra-articular injection

Artificial miRNA-containing plasmids targeting murine *Shn3* were generous gifts from Dr. Guangping Gao. Replication-deficient recombinant AAV (rAAV) vector design and production were performed as previously described^35^. In brief, engineered amiR cassettes targeting Shn3 (*amiR-Shn3*) or control (*amiR-ctrl*) were constructed within vector plasmids between *CB* promoter and the reporter gene *Egfp* which enable visual tracking of transduced cells, or tissues. Plasmids containing amiR cassettes, AAV2/9 and helper plasmids were mixed and transfected into HEK293 cells using PEI-MAX 40000 (Polysciences, 24765–1) to generate rAAV for experimental use. These rAAV batches were then collected and purified following a traditional CsCl sedimentation protocol. The concentration of rAAV9-*amiR-Shn3* and rAAV9-*amiR-ctrl* was then tittered as previously described (Yang YS, Nat Commun, 2020).

For local delivery of rAAV, intra-articular injections were performed when male- *Col1a2*^*oim/oim*^ mice or controls reached 1 month of age. To avoid confounding due to active fracture repair, *Col1a2*^*oim/oim*^ mice display radiographic evidence of fracture were excluded from the experimental cohort. After anesthesia and surgical site preparation, a 1-mm anterolateral skin incision was made above knee articular capsule. A total volume of 5uL containing 1 × 10^12^ GC rAAV9-*amiR-shn3* or rAAV9-*amiR-ctrl* were injected into the articular capsule of contralateral hindlimb stifle/knee joints on the same host to allow for paired analysis of local effects in the same host. The needle was retained in the injection site for 2–5 minutes to avoid leakage. Incisions were then sutured and analgesic administration was performed as described previously^14^. 8 weeks after injection, individuals were subjected to in vivo IVIS optical imaging and lower limbs were dissected for downstream analysis.

### Statistical analysis

All statistical analysis was performed using GraphPad Prism (v6.0a; GraphPad, La Jolla, CA, USA). A two-tailed Student’s t test was used to determine significance for comparison of only two groups. One-way ANOVA with Tukey’s post-hoc tests were used to determine significance for comparisons between multiple groups. A P value < 0.05 indicated statistical significance. Error bars are presented as mean ± s.e.m.
